# A pooled analysis of injection site-related adverse events in patients with schizophrenia treated with olanzapine long-acting injection

**DOI:** 10.1186/1471-244X-14-7

**Published:** 2014-01-14

**Authors:** Susan Atkins, Holland C Detke, David P McDonnell, Michael G Case, Shufang Wang

**Affiliations:** 1 Lilly Research Laboratories, Eli Lilly and Company, Lilly Corporate Center, Indianapolis, IN 46285, USA

**Keywords:** Olanzapine long-acting injection, Schizophrenia, Adverse events, Injection site reactions, Pamoate

## Abstract

**Background:**

Depot antipsychotic injections are an important tool for the management of patients with schizophrenia who have difficulty with adherence to oral medication. However, pain and discomfort at the injection site can be a potential impediment to the use of these long-acting formulations. We report here the results of a pooled analysis of injection site-related adverse events (AEs) collected during treatment with the olanzapine long-acting injection (olanzapine LAI).

**Methods:**

Unsolicited injection site-related AEs were pooled from 7 olanzapine LAI clinical trials conducted in patients between March 2001 and December 2010. All patients had a *Diagnostic and Statistical Manual of Mental Disorders, Fourth Edition* (DSM-IV) *or Fourth Edition, Text Revision* (DSM-IV-TR) diagnosis of schizophrenia or schizoaffective disorder and were between the ages of 18 and 75. Doses ranged from 45 to 405 mg olanzapine LAI, and injection intervals were 2, 3, or 4 weeks. Events were evaluated for severity, timing, possible risk factors, and outcome. A criterion of *p* < .05 for statistical significance was used for all tests.

**Results:**

A total of 1752 patients received at least 1 olanzapine LAI injection. Of these, 92 patients (5.3%) reported at least 1 injection site-related AE, with “pain” being the most common type (2.9%). Most events were mild (81.4%) and the median duration was 3 days. Four patients (0.2%) discontinued due to injection site-related AEs. Dose volume and body mass index did not appear to affect the probability of injection site-related AEs. However, patients who experienced a post-injection delirium/sedation syndrome event (n = 37) were more likely to have or have had an injection site-related AE at some time during the study. Incidence of injection site-related AEs appeared to decrease over time. In 94.2% of the injection site-related AEs, no specific treatment or concomitant medication was reported; in 9 cases, patients received pharmacologic treatment for reaction, mass, abscess, rash, or pain.

**Conclusions:**

Injection site-related AEs with olanzapine LAI were generally mild. The incidence and nature of these injection site-related AEs were generally similar to those occurring during treatment with other injectable antipsychotics.

**Trial registration:**

ClinicalTrials.gov ID; URL: NCT00094640, NCT00088478, NCT00088491, NCT00088465, and NCT00320489.

## Background

Despite advances in the treatment of schizophrenia with the use of newer atypical antipsychotic agents, treatment nonadherence continues to be a problematic and costly issue. As many as one-third of outpatients are reported to be completely noncompliant, and another third are reported to be only partially compliant [1]. Moreover, patient adherence has been found to worsen over time, with up to 75% of patients demonstrating only partial or worse compliance after 2 years of treatment [2].

Long-acting depot injections of antipsychotic agents are important in the treatment of schizophrenia because they may help mitigate treatment noncompliance and its consequences. However, pain and discomfort at the injection site can be a potential impediment to the use of these depot formulations. Injection site adverse events (AEs), including injection site pain, have been reported with conventional and atypical depot agents [3-6]. However, the extent of injection site-related AEs reported with injectable long-acting antipsychotic medications is not widely studied in the literature. Rates as low as 1.9% [7] and as high as 10% [8] have been reported in different papers.

Olanzapine long-acting injection (LAI) is a depot antipsychotic formulation consisting of a pamoate salt of olanzapine that is administered by deep intramuscular injection every 2 to 4 weeks [9]. The efficacy and safety of olanzapine LAI have been established in the treatment of schizophrenia in several reports [10-12]. In addition, the efficacy and safety of olanzapine LAI have been shown to be similar to that of oral olanzapine [10], with the exception being incidence of local injection site-related AEs and post-injection delirium/sedation syndrome (PDSS) events. PDSS events, characterized by signs of delirium and/or excessive sedation consistent with symptoms of olanzapine overdose, were identified during clinical studies and occurred in 0.07% of injections and in approximately 1.4% of patients [13]. Because of the risk of PDSS, safety precautions outlined in the approved label—including a post-injection observation period—must occur at the time of each injection [9].

The purpose of this paper is to determine the extent and nature of injection site-related AEs in patients treated with olanzapine LAI. An analysis of pooled data from 7 olanzapine LAI studies will be presented.

## Methods

### Studies

This pooled analysis is based on 7 olanzapine LAI clinical trials that were conducted in patients between March 2001 and December 2010: a single-dose pharmacokinetic study (*N* = 134, Study F1D-EW-LOBS), a 2-month pharmacokinetic study (*N* = 9, Study F1D-EW-LOBO), a receptor occupancy study (*N* = 14, Study F1D-EW-HGJW) [14], an 8-week randomized, placebo-controlled acute efficacy study (olanzapine LAI *N* = 304, Study F1D-MC-HGJZ) [11], a 24-week randomized, oral olanzapine-controlled maintenance study (olanzapine LAI *N* = 743, Study F1D-MC-HGKA) [10], a 2-year randomized, oral olanzapine-controlled open-label efficacy study (olanzapine LAI *N* = 264, Study F1D-MC-HGLQ), and a 6-year open-label extension study (*N* = 931, Study F1D-MC-HGKB) [12]. In addition there was 1 trial (Study F1D-EW-LOBE, a 6-month pharmacokinetic study) that was not included in this analysis due to the fact that the injection-related AEs for that study were collected through active solicitation (27.8% of patients [*N* = 302] reporting an injection site-related AE) and thus were not comparable with those of the other studies, which relied solely upon spontaneous reporting of AEs.

### Subjects

All patients had a diagnosis of schizophrenia or schizoaffective disorder, as defined by the *Diagnostic and Statistical Manual of Mental Disorders, Fourth Edition* (DSM-IV) *or Fourth Edition, Text Revision* (DSM-IV-TR), were between the ages of 18 and 75, and had at least 1 olanzapine LAI injection [13]. Exclusion criteria included significant suicidal or homicidal risk; pregnancy or breastfeeding; acute, serious, or unstable medical conditions; or substance dependency (except nicotine or caffeine) within the past month.

### Olanzapine LAI injection procedures

Patients received their injections after completion of all efficacy and safety assessments at that visit. Injections were generally administered into alternating sides of the buttocks from visit to visit, and administrators were advised not to massage the injection area after injection. Injections were administered using a 19-gauge 1.5-inch (or 35-mm) needle; a 2-inch (or 50-mm) needle could be used for obese patients. Doses ranged from 45 to 405 mg olanzapine LAI, and injection intervals could be 2, 3, or 4 weeks, depending on the specific study. Injections given at 2-week intervals could not exceed a dose of 300 mg.

### Statistical analyses

All analyses were performed using SAS version 9.1 (SAS Institute, Inc., Cary, N.C.). Only injection site-related AEs were assessed (i.e., AEs with Medical Dictionary for Regulatory Activities [MedDRA] preferred terms that contained the phrase “injection site”). The Cochran-Armitage test for trend was used to assess whether there was any increasing or decreasing trend in the relationship between the presence of injection site-related AEs and injection volume (specific doses were 45, 135, 150, 210, 225, 255, 270, 300, 330, 345, 360, 375, and 405 mg), or body mass index (BMI; categories were underweight [BMI < 18.5]; normal weight [BMI ≥18.5, <25]; overweight [BMI ≥25, <30]; and obese [BMI ≥30: 32/510]). Because a patient’s BMI category could change over the course of a study, BMI at the first injection was used for patients who never reported an injection site-related AE, but for patients with injection site-related AEs BMI at the first occurrence was used. Chi-square tests were used to compare any 2 proportions. Fisher’s Exact test was used to compare the percentage of patients experiencing both PDSS and at least one injection site-related AEs with the percentage of patients who experienced PDSS only. A criterion of *p* < .05 for statistical significance was used for all tests.

## Results

From the 7 pooled studies, a total of 1752 patients received at least 1 olanzapine LAI injection (Total = 56,240 injections). Of these 1752 patients, 92 (5.3%) reported at least 1 injection site-related AE (Table 1). There was a total of 156 injection site-related AEs. Table 1 also presents events that occurred more than once for patients. Out of 92 patients who experienced an injection site-related AE, 19 (20.7%) experienced the same event more than once. Of the patients who experienced an injection site-related AE, a substantial percentage (42.4%) experienced pain only and no other type of event.

**Table 1:** Percentages of patients with injection site-related adverse events

**Preferred term**	**Patients with 1 or more event (N = 1752) n (%)**	**Patients with events occurring more than once n (%)**
Any injection site-related AE	92 (5.3)	19 (20.7)
Injection site pain	50 (2.9)	6 (12.0)
Injection site reaction	10 (0.6)	1 (10.0)
Injection site mass	8 (0.5)	3 (37.5)
Injection site swelling	8 (0.5)	3 (37.5)
Injection site induration	6 (0.3)	1 (16.7)
Injection site nodule	6 (0.3)	2 (33.3)
Injection site irritation	5 (0.3)	2 (40.0)
Injection site abscess	4 (0.2)	
Injection site haemorrhage	4 (0.2)	1 (25.0)
Injection site warmth	3 (0.2)	
Injection site erythema	2 (0.1)	
Injection site discolouration	1 (0.1)	
Injection site extravasation	1 (0.1)	
Injection site oedema	1 (0.1)	
Injection site paraesthesia	1 (0.1)	
Injection site rash	1 (0.1)	1 (100.0)

*Abbreviation: AE* adverse event.

All injection site-related AEs were classified by the investigator as mild, moderate, or severe: 81.4% of the events were considered mild, 17.3% were of moderate severity, and 2 events (1.3%, both injection site reactions) were severe. Four patients (0.2%) discontinued due to injection site-related AEs (1 each of injection site reaction [severe], nodule [moderate severity], swelling [moderate severity], and a mass [mild severity]). None of these subjects had experienced a previous event.

There was no significant relationship between dose volume and the probability of an injection site-related AE (*p =* .149).

An analysis of injection site-related AEs and PDSS events demonstrated that the percentage of patients who experienced a PDSS event was greater among those who, at some point during the study, also experienced an injection site-related AE versus those who did not (5/92 = 5.4% vs. 32/1660 = 1.9%; Fisher’s Exact test *p =* .041).

We also tested if the percentage of patients experiencing an injection site-related AE was associated with BMI. The percentages of patients in each of the BMI categories who experienced an injection site-related AE were as follows: underweight (6.1%); normal weight (4.5%); overweight (5.1%); and obese (6.3%). BMI category was not significantly associated with percentage of injection site-related AEs (Cochran-Armitage test for trend, *p =* .254).

**Figure 1:**
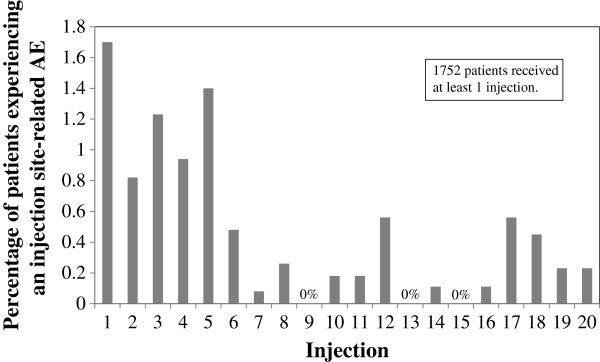
**Timing of injection site-related adverse events, by injection.** Abbreviation: AE = adverse event.

The timing of injection site-related AEs over the course of treatment is shown in Figure 1. The percentage of events appears to decrease with successive injections.

The durations of injection site-related AEs are shown in Table 2. It should be noted that 1 data point was excluded from the data analysis because it was an extreme outlier and most likely an error (1097 days of injection site pain, which was greater than 300 times the median). The median duration of all occurrences of all event types for which duration was known was 3 days. The largest category of AE was injection site pain, which had a median duration of 1.5 days. Durations of 18 events are missing because they were not recovered, were recovering/resolving, or were unknown at the time the patient discontinued the study.

**Table 2:** Duration of injection site-related adverse events (combined and separately for injection site pain)

	**N**	**Mean**	**SD**	**Min.**	**25**^**th **^**Percentile**	**Med.**	**75**^**th **^**Percentile**	**Max.**
**All events**								
Duration of all occurrences, days	137	9.7	16.9	1	1	3	11	117
Duration of first occurrence, days	98	10.0	14.9	1	1	3	14	86
**Injection site pain**								
Duration of all occurrences, days	68	3.6	5.0	1	1	1.5	3.5	32
Duration of first occurrence, days	48	4.4	5.7	1	1	2.5	4.5	32

*Abbreviations: Max* maximum, *Med* median, *Min* minimum, *SD* standard deviation.

Note: One data point was excluded because it was an extreme outlier. Durations are not known for 18 events.

Medications were provided when necessary for the treatment of injection site-related AEs, but in most cases (83/92 patients [90.2%]; 147/156 events [94.2%]) no concomitant medications were administered (or at least captured and tied specifically to the event). However, concomitant medications *were* given to 9 patients with injection site-related AEs. Cefalexin was given to 5 patients for diverse events (2 patients with abscesses, 2 with injection site reactions, and 1 with a mass), hydrocortisone was given to 1 patient with a mass, amoxicillin was given to 1 patient with an injection site reaction, sodium metamizole was given to 1 patient for pain, and betamethasone was given to 1 patient with a rash.

## Discussion

The safety and efficacy of olanzapine LAI have been reported previously and have been shown to be similar to those of oral olanzapine [10-12], with the exception being the occurrence of injection-related AEs. Injection-related AEs for olanzapine LAI can include AEs localized to the injection site itself but can also include PDSS events [13,15]. The purpose of the present analysis was to focus specifically on injection site-related AEs, which are not well understood but may have an impact on patient satisfaction and treatment adherence.

Injection site-related AEs occurred in a small percentage of patients (5.3%). When these did occur, most were identified as mild (81.4%), with a median duration of 3 days. Pain was the most common type of event, occurring in 2.9% of patients, but each of the other types of injection site-related AEs occurred in fewer than 1% of patients. Dose volume and BMI did not appear to affect the probability of injection site-related AEs. The percentage of patients who experienced a PDSS event was greater among those who, at some point during the study, also experienced an injection site-related AE versus those who did not (5.4% vs. 1.9%, *p =* .041). This may be related to unknown factors at the injection site that may make these patients at higher risk for both events and needs to be further explored. However, it should be noted that this relationship is based on a small number of patients with an event (*n* = 37) and that these shared risk factors imply only an association between the injection site-related AEs and PDSS and that these events are not necessarily causally related.

There was some evidence that the likelihood of an event decreases with the number of injections received. This is somewhat expected, however, as patients who are not susceptible to injection site events will tend to stay on the medication longer. In addition, this trend could be enhanced by the decreased reporting of certain events, such as pain or irritation, over time as the patient becomes acclimated to the injections.

The most common injection site-related AE was pain (2.9% of patients). In a very few instances, the pain resulted in the prescription of an analgesic. The other medications prescribed were antibiotics or an anti-inflammatory, prescribed for injection site reaction, mass, abscess, or rash. There were very few discontinuations due to injection site-related AEs.

The incidence of injection site-related AEs in the present analysis was similar to that found in previous studies of injectable neuroleptics. In a recent review of a small number of olanzapine LAI studies, Cañas and Möller [16] noted an injection site AE rate of 3.6%. Hamann et al. [4] found that 7.7% of subjects experienced an injection site reaction after administration of haloperidol decanoate. Citrome [8] recently reviewed 6 studies of paliperidone palmitate and found rates of injection site reactions of 4% to 10%. Quiroz et al. [7], reporting the results of 2 studies of risperidone LAI, found the incidence of injection site-related AEs was ≤2% (Study 1, *N* = 170). In the second study (*N* = 53) injection site pain and injection site reaction were reported as AEs by 7.5% and 1.9% of patients, respectively. Thus, the 5.3% incidence of injection site-related AEs observed with olanzapine LAI appears within the same range as that observed with other injectable antipsychotics, despite the use of a larger needle size and a larger volume of medication being injected with olanzapine LAI.

It is not possible to avoid all local injection site-related AEs, but some may be prevented by proper injection techniques [17,18]. Some important considerations include having aseptic conditions and keeping patients relaxed during injection (avoiding sudden movement). There is also the potential that pain can be reduced by ensuring that the medication is at room temperature at time of injection, giving the injection using the right momentum (as forcing too much medication into the muscle too quickly could contribute to a greater risk of ‘tearing’ of muscle fibers resulting in trauma at site of injection), and using comfort measures, such as the application of heat or cold to the injection site [19]. Jones et al. [5] also found that decreasing the size of injections (more concentrated) and the frequency of injections reduced the rate of injection site-related AEs. Olanzapine LAI does not require refrigeration and should be injected with steady, continuous pressure [9]. Although it is not recommended to massage the site after the injection, use of a cold compress is an acceptable comfort measure [19]. Olanzapine LAI is only offered at 1 concentration; however, dosing and injection frequency can be modified to decrease injection frequency to monthly if needed. A needle smaller than 19 gauge should not be used as this could increase the risk of needle clogging [9].

### Limitations

The authors recognize several limitations of this report and the studies from which the data were drawn. First, although the majority of the injection site-related AEs were injection site pain, none of the studies included a formal assessment of pain severity. Second, some patients reported injection site-related AEs as ongoing at the time of discontinuation from the study, and thus it is not possible to know the outcome of these events. Finally, our event-term search strategy required “injection site” to be specified in the event term; however there may be other events in the database which were related to the injection but were excluded because the location of the reaction was not specified.

## Conclusions

For patients treated with olanzapine LAI, injection site-related AEs were generally mild. Pain was the most common type of event, occurring in 2.9% of patients, but each of the other types of injection site-related AEs occurred in fewer than 1% of patients. The incidence and nature of these injection site events were generally similar to those occurring during treatment with other injectable antipsychotics. Importantly, the rate of discontinuation due to injection site-related AEs was low, suggesting that the risk of these adverse events will not change the benefit/risk profile for most patients considering treatment with depot medications.

## Competing interests

Ms. Atkins, Dr. Detke, Dr. McDonnell, Mr. Case, and Dr. Wang are employees and minor stockholders of Eli Lilly and Company.

## Authors’ contributions

All of the authors participated in the drafting or critical review of this manuscript. MC and SW were primarily responsible for the statistical analysis. SA was primarily responsible for interpretation of one or more of the studies that were included in this report. HD and DM were primarily responsible for the planning and/or design of one or more of the studies that were included in this report. All authors read and approved the final manuscript.

## Pre-publication history

The pre-publication history for this paper can be accessed here:

http://www.biomedcentral.com/1471-244X/14/7/prepub
